# Shear behavior of high-strength reinforced concrete beams with circular openings under fire exposure

**DOI:** 10.1038/s41598-026-43162-y

**Published:** 2026-04-22

**Authors:** Ahmed E. Sedawy, Ashraf A. A. Beshr, Islam Ali Mahmoud

**Affiliations:** 1 Civil Engineering Department, Misr Higher Institute for Engineering and Technology, Mansoura, Egypt; 2 Public Works Department, Faculty of Engineering, Mansoura University, Mansoura, 35516 Egypt; 3 Civil Engineering Department, Delta Higher Institute for Eng. & Technology, Mansoura, Egypt

**Keywords:** High-strength concrete, Fire exposure, Circular openings, Steel fibers, Ferrocement retrofitting, Engineering, Materials science

## Abstract

This study investigates the shear behavior of high-strength concrete (HSC) beams with large circular web openings subjected to elevated temperatures. Internal (steel fibers at 0.5% and 1.0% by volume) and external (ferrocement jackets) retrofitting techniques were evaluated. Nine beams were experimentally tested, including solid control specimens, un-strengthened beams, and retrofitted beams. Exposure to 500 °C resulted in a shear capacity reduction of up to 68% for beams with 150 mm openings. After thermal exposure, steel fibers enhanced the residual shear strength by up to 16.4%, while ferrocement jackets achieved recovery levels of up to 14.5%. A parametric finite element study was conducted considering opening diameters ranging from 100 to 200 mm and temperature levels between 400 and 600 °C, demonstrating strong agreement with experimental results (average deviation ≤ 3%). The findings provide validated insights into the combined effects of geometric discontinuities and thermal degradation and support the development of effective retrofitting strategies for HSC beams under fire conditions.

## Introduction

One of the main reasons web openings are introduced in reinforced concrete (RC) beams is to provide a passage for mechanical and electrical services or to reduce the story height by eliminating unused space. Over the past decades, numerous studies have examined the structural behavior of beams with various opening shapes, including rectangular, circular, diamond, triangular, trapezoidal, and irregular forms^1^.

Circular openings are generally considered large when their diameter exceeds 25% of the web depth of the beam^2^. In beams subjected to simultaneous bending and shear, increasing the opening size alters the internal load transfer through Vierendeel truss action, particularly for members with a low span-to-depth ratio^3,4^. Salam et al. reported that beams with small openings in pure bending generally maintain their ultimate load capacity^5,6^, and observed failure modes are similar to solid beams when sufficient shear reinforcement is provided^7^. In contrast, large openings significantly disrupt internal stress flow, causing the regions above and below the opening to act as compression struts and tension ties, which can lead to premature failure^8,9^.

Steel fibers in concrete enhance mechanical performance, especially shear strength and post-cracking resistance^10–13^. Fiber-reinforced concrete (FRC) exhibits higher tensile capacity, ductility, and energy absorption. The combined action of steel fibers with longitudinal bars and stirrups can elevate both shear and flexural strength. Deep fiber reinforcement can increase shear strength by up to 20% compared to conventional RC beams^14^. Ferrocement is another effective retrofitting alternative, known for its ease of application and potential to strengthen members without requiring special bonding agents or surface preparation^16,15^.

High temperatures severely degrade concrete properties. The thermal damage zone is often limited to the outer 30–50 mm of the cross-section, while strength reduction and cracking may threaten the global load-carrying capacity^17^. The addition of 1.0% steel fibers can increase residual compressive strength by up to 16.5% after exposure to 500 °C and also enhance the elastic modulus by around 29% under normal conditions^18^.

While the literature is extensive regarding RC beams with web openings and various strengthening methods, only a few studies have addressed the combined effect of fire exposure and retrofitting on high-strength concrete (HSC) beams. HSC, defined as concrete with a compressive strength above 60 MPa, is known for high durability and low permeability, but its brittleness and susceptibility to explosive spalling at elevated temperatures remain critical challenges.

The introduction of web openings facilitates mechanical and electrical services but reduces shear capacity, particularly under high temperatures. Prior studies have explored these effects^2,18^ (Kodur & Agrawal, 2016; Mansur & Tan, 1999), and various retrofitting techniques such as steel fibers and ferrocement have been investigated. However, few studies examine the combined effect of fire exposure and retrofitting on HSC beams with large circular openings, particularly comparing internal steel fiber and external ferrocement strategies. This study fills this gap by providing experimental and FEM validation, investigating post-fire performance, and offering parametric insights for structural safety.

Recent studies have highlighted the vulnerability of RC and HSC members with openings when subjected to elevated temperatures, emphasizing the need for reliable post-fire assessment and strengthening strategies^19–22^. However, these studies primarily focused on either fire exposure or strengthening techniques independently, without providing a comparative evaluation of internal and external retrofitting methods for HSC beams with large circular web openings.

## Significant research

A large amount of investigation has been done to study the response of reinforced concrete (RC) beams with openings in the web, the main issues addressed by the researchers being the strength reduction, the development of cracks, and the mechanisms of failure. It has been found that circular openings beyond 25% of the web depth cause a major decrease in shear capacity and stiffness. Mansur and Tan found that openings located near the mid-span of a beam mainly affect the flexural behavior of the beam, while openings closer to the supports lead to shear resistance being compromised most. Moreover, the fire performance of the RC beams has been thoroughly studied as well. Khoury^23^ and Hertz^24^ provided evidence that raised temperatures lead to a decrease in compressive strength, stiffness, and bond integrity between reinforcing steel and the surrounding concrete. These changes become progressively severe as the strength of the concrete increases due to the dense microstructure and low permeability that, consequently, make the concrete more susceptible to explosive spalling and brittle failure when exposed to high temperatures^4^.

Many methods have been introduced to improve the function of the reinforced concrete (RC) beams with openings. Among them, externally applied techniques like ferrocement jacketing, wrapping with fiber-reinforced polymer (FRP), and steel plate bonding have received great attention due to the shear and flexural capacity restoration. In addition to these, ferrocement can be regarded as one of the most effective ways as a result of the low cost, the easy installation, the crack controlling capability, and the enhancement of post-cracking response^5–7^.

Furthermore, the use of internal strengthening by steel fiber addition has been instrumental in the rise of ductility, energy absorption, and fire resistance of reinforced concrete members, in particular, when used in conjunction with high-strength concrete^8–10^. At the same time, some unanswered questions, however, still linger in the scientific literature regarding the joint impact of fire exposure and retrofitting on web HSC beams with openings. Most of the investigations reported by the authors are confined only to ordinary concrete, whereas the present problem deals with the coupled thermal–mechanical effects in HSC beams weakened by large circular openings. Besides this, there is a scarcity of comparative studies that have systematically assessed the efficiency of internal strengthening with discrete steel fibers as compared to external retrofitting with ferrocement under temperature.

Therefore, the present work fills in the only gap that remains by experimentally verifying the shear performance of high-strength concrete beams with circular openings exposed to fire. It is a comparative study of two different methods of retrofitting: (i) internal strengthening through the incorporation of steel fibers and (ii) external retrofitting by way of the application of a ferrocement jacket. The results are likely to indicate the degree of effectiveness of these means in the return of the lost by combined thermal and mechanical loading strength, stiffness, and ductility.

## Experimental program

**Fig. 1:**
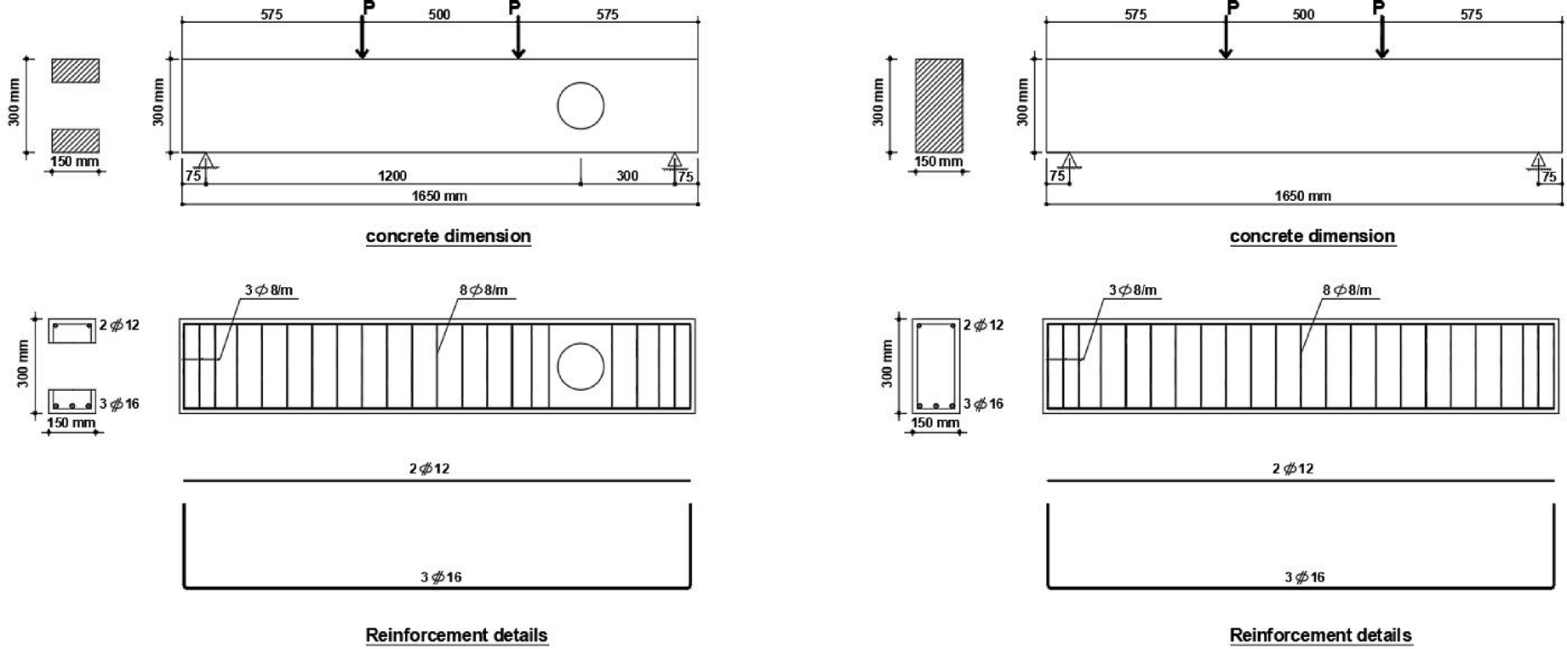
Details of typical specimen.

Nine high-strength reinforced concrete (HSC) beams were cast and tested to investigate the structural performance of beams with circular web openings subjected to elevated temperatures, and to assess the effectiveness of internal and external retrofitting techniques. All beams shared identical rectangular cross-sections of 150 mm width × 300 mm depth and a total length of 1600 mm. For specimens containing web openings, the opening diameter was fixed at 150 mm. The general configuration of the tested specimens is illustrated in Fig. 1. A consistent labeling system was adopted, consisting of a group designation followed by a specimen number. The key variables investigated in this study include the presence of circular web openings, fire exposure (up to 500 °C), internal strengthening using discrete steel fibers (at 0.5% and 1.0% volume fractions), and external retrofitting using ferrocement jackets.

Table 1 gives a summary of the different specimen configurations and the parameters of the test. The specimens were divided into four main groups. The first group consisted of three beams: a solid control beam without any openings (B1), and two beams with circular web openings (B2 and B3). Of these, B3 was exposed to fire at 500 °C and then water-cooled to simulate post-fire conditions. The second group explored the impact of internal strengthening by using discrete steel fibers. Beams B4 and B5 were reinforced internally with 0.5% and 1.0% steel fibers by volume, respectively, and tested without fire exposure. Beams B6 and B7 had the same internal strengthening but were exposed to elevated temperatures followed by rapid cooling.

**Table 1:** The Experimental Program & Results.

Group	Specimen	Opening dimension (mm)	Strengthen material	Fire condition	First crack load Pcr(KN)	Failure load Pu (KN)	Pu/Pu(control)	Deflection at P_max_	Max deflection/ Max-deflection (control)
G1	**B1**	**–**	**–**	No Fire	**232.2**	**666**	**1**	**15.18**	**1**
	B2	Ø150	**–**		81.72	216	0.32	8.11	0.53
	B3	Ø150	**–**	Fire	72	175	0.26	7.125	0.47
G2	B4	Ø150	Steel fiber 0.5%	No Fire	82.26	228	0.34	6.55	0.43
	B5	Ø150	Steel fiber 1.0%		138.96	237	0.36	2.61	0.17
	B6	Ø150	Steel fiber 0.5%	Fire	72	187	0.28	7.02	0.46
	B7	Ø150	Steel fiber 1.0%		72	203	0.3	5.96	0.39
G3	B8	Ø150	Ferrocement	No Fire	56	237.5	0.36	3.52	0.23
	B9	Ø150	Ferrocement	Fire	72	200.87	0.3	4.935	0.33

The third group was centered around external retrofitting with the application of ferrocement layers. Before the test, B8 was strengthened on the outside around the web opening. The B9 was similarly retrofit but it was first subjected to fire exposure at 500 °C, then water-cooled, and only then tested.

Each concrete mixture was a high-strength one, with the aim of the compressive strength being more than 60 MPa. The mix was made of Ordinary Portland Cement (OPC), natural silica sand, crushed dolomite, and potable water. As for the steel fibers in the beams (B4 to B7), the corrugated steel fibers with a thickness of 0.5 mm, a width of 3 mm, and a length of 50 mm were adopted. The compressive strengths measured were 67 MPa and 71 MPa for 0.5% and 1.0% fiber contents, respectively.

The external retrofitting of B8 and B9 were carried out by using a ferrocement layer consisting of two layers of 14 mm square wire being symmetrically arranged on both sides of the opening. To avoid the steel shear connectors that were used for anchoring the mesh from being detached, the mesh was embedded in a cement–sand mortar with a 1:2 mix ratio and was fixed with steel shear connectors.

To create circular openings, PVC tubes (150 mm diameter) were placed before casting. Fire exposure was given to beams B3, B6, B7, and B9. The specimens were heated to 500 °C and kept at that temperature for one hour. The fire exposure was discontinued after one hour for the fire-exposed beams, and the beams were cooled with a water jet for 15 min, followed by one hour of air cooling., All the beams were moist-cured under wet burlap for 28 days. After curing and fire exposure (if any), four-point bending tests were performed to determine the load-carrying capacity, deflection features, and failure mechanisms. The Table 1 shows all the details of tested beams with different parameters.

## Experimental setup and testing

**Fig. 2:**
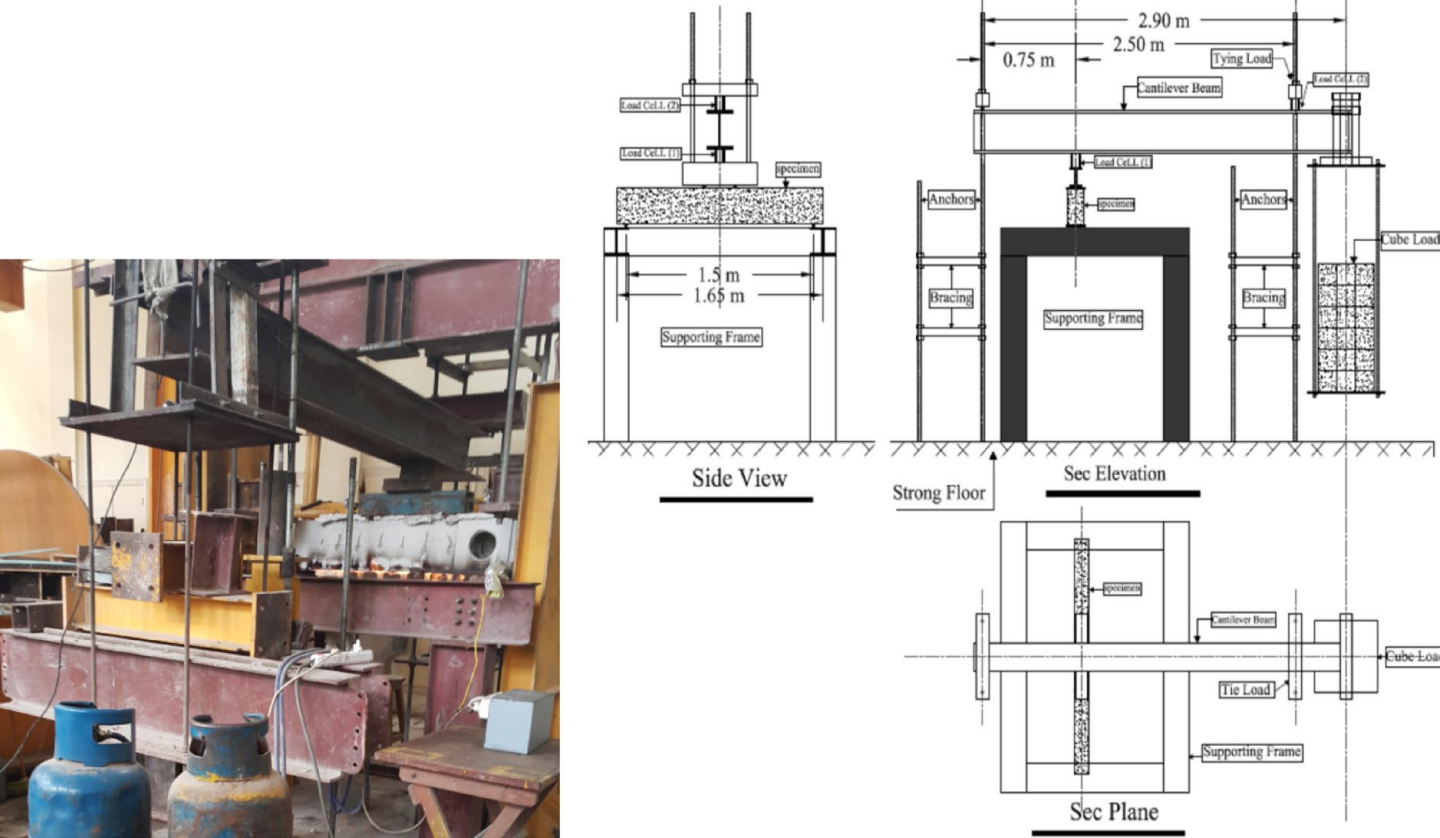
Sketch of experimental test setup.

Every experimental procedure was conducted at the Helwan University-Maria Branch Structural Engineering Laboratory of the Faculty of Engineering. As shown in Fig. 2, a specially made steel test frame was built to enable both structural loading and fire exposure. Four vertical steel columns held up steel I-beams, creating a rigid frame with a clear span of 1500 mm between beam supports.

A secondary I-section beam that served as a lever mechanism was incorporated into the loading system. One end of the lever was fastened to the sturdy floor of the lab, and the other end was suspended from a steel hanger with calibrated steel weights. Accurate load measurements were ensured by using a precision load cell to transfer the load through the lever to a central loading point above the tested beam.

Steel weights that were suspended up to 30 kN were used to gradually apply the initial loading. At this point, beams designated for fire testing were subjected to 500 °C for an hour while supporting the applied load, then cooled with water for 15 min and rested for another hour. The displacement-controlled loading protocol was restarted after cooling, enabling incremental load increases until failure. The displacement-controlled protocol was implemented immediately following the initial 30 kN load for beams that were not exposed to fire. By eliminating the need for hydraulic jacks during heating, this setup increased operational safety and guaranteed accurate test conditions.

The digital load cell, which was linked to a real-time data acquisition system, had a 1200 kN capacity and an accuracy of ± 0.1 kN. Using three Linear Variable Differential Transformers (LVDTs), two of which were placed 300 mm from each support and one at mid-span, vertical deflections were measured for beams that were not exposed to fire. To prevent thermal sensitivity problems with the electronic sensors, mechanical dial gauges were placed in the same places for beams exposed to fire.

## Test results and analysis

**Fig. 3:**
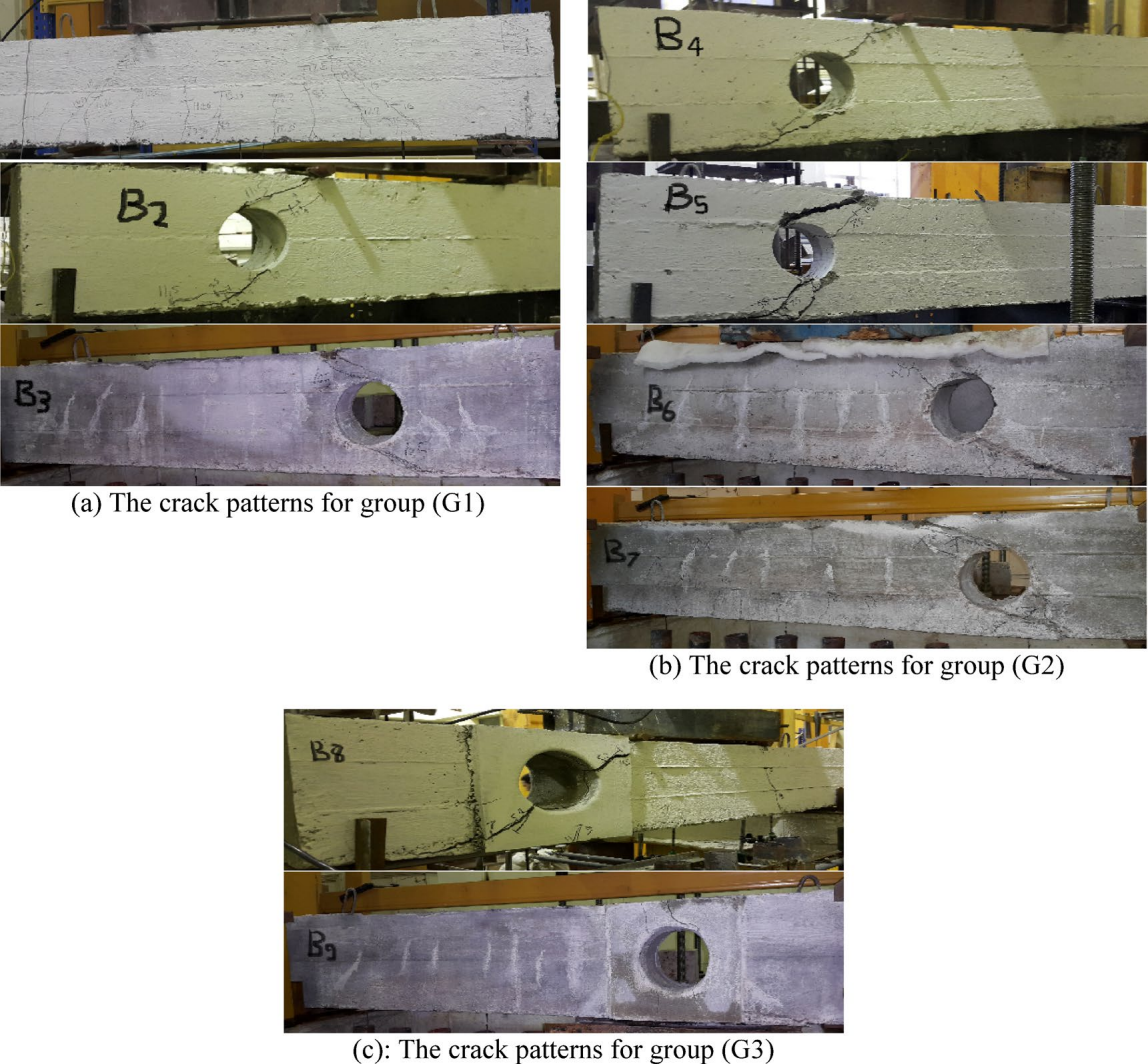
The crack patterns of tested specimens.

**Fig. 4:**
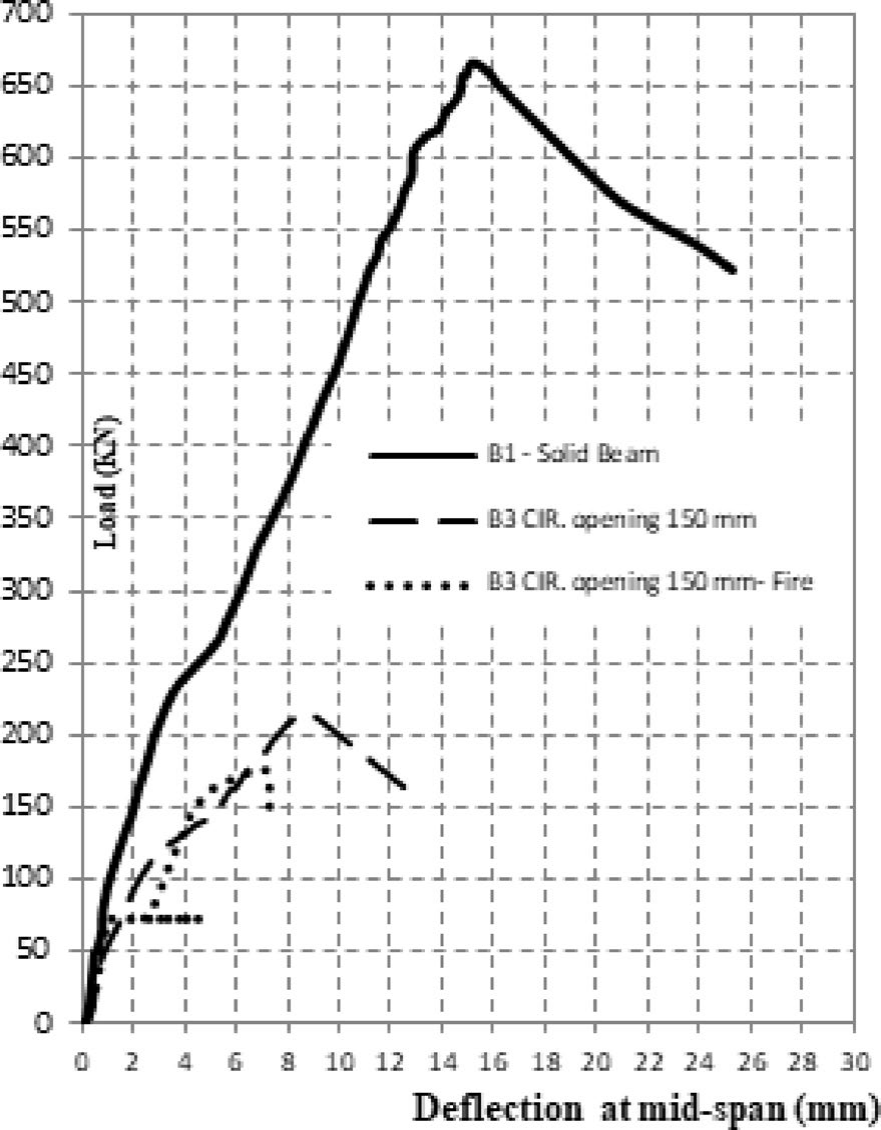
Effect of fire on Load–deflection for an open beam.

**Fig. 5:**
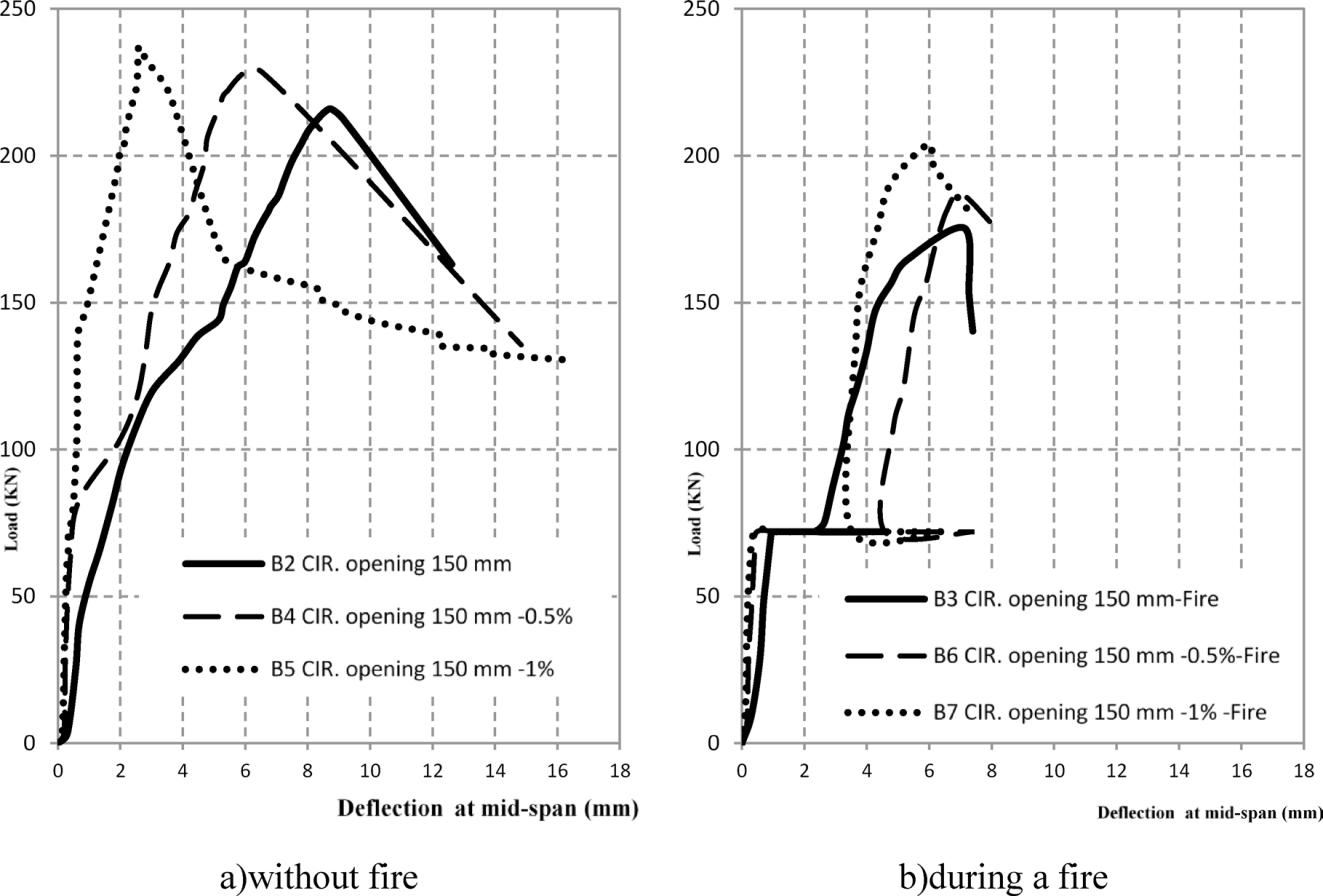
Load–deflection for retrofitted beams by discrete steel fiber.

**Fig. 6:**
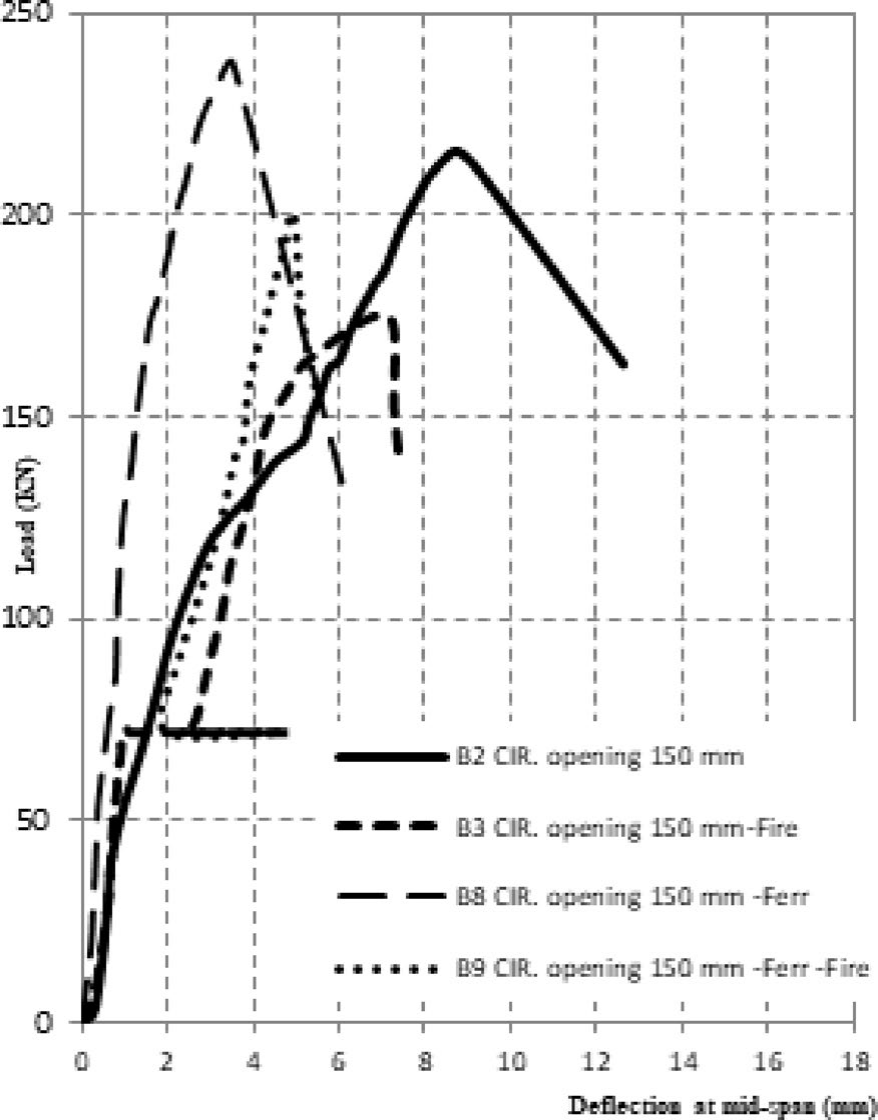
Load–deflection for retrofitted beams by ferrocement.

The experimental results of the tested high-strength reinforced concrete (HSC) beams are presented in Tables 1 and 2 and illustrated through Figs. 3, 4, 5, 6. Table 1 summarizes the first cracking load, ultimate load, and maximum deflection values, while Table 2 provides ductility indices and energy absorption capacities. Together, these results highlight the structural performance of beams with circular web openings under different conditions, including exposure to elevated temperatures and the use of internal and external strengthening techniques.

**Table 2:** the ductility index and area energy absorption of tested RC beams.

Group	Specimen	Opening dimension (mm)	Strengthen material	Fire condition	∆Max	∆failure	Ductility index = ∆ailure /∆Max	Area under P-d	Reduction of area
G1	**B1**	**–**	**–**	No Fire	**15.18**	**25.28**	**1.67**	**7502**	1
	B2	Ø150	**–**		8.11	12.624	1.56	1890	0.25
	B3	Ø150	**–**	Fire	7.125	9.435	1.32	1005	0.13
G2	B4	Ø150	Steel fiber 0.5%	No Fire	6.55	14.85	2.27	1980	0.26
	B5	Ø150	Steel fiber 1.0%		2.61	16.56	6.34	2250	0.3
	B6	Ø150	Steel fiber 0.5%	Fire	7.02	9.08	1.29	1060	0.14
	B7	Ø150	Steel fiber 1.0%		5.96	7.4	1.24	1159	0.15
G3	B8	Ø150	Ferrocement	No Fire	3.52	6.01	1.71	1020	0.14
	B9	Ø150	Ferrocement	Fire	4.935	5.57	1.13	793	0.11

The analysis focuses on important response parameters like failure modes, crack initiation and propagation, and load–deflection behavior. All specimen groups were compared in order to evaluate the effects of the following: the presence of circular web openings; exposure to fire at 500 °C followed by cooling; internal strengthening with discrete steel fibers (0.5% and 1.0% by volume); and external retrofitting with ferrocement jackets.

The comparative evaluation demonstrates the influence of each variable on flexural capacity, stiffness degradation, ductility, and failure mechanisms. These insights form the basis for understanding how strengthening strategies can mitigate the detrimental effects of openings and fire exposure on the performance of high-strength RC beams.

### Crack patterns and modes of failure

The crack patterns and failure modes of the high-strength reinforced concrete (HSC) beams are shown in Fig. 3. For the solid control beam (B1), both flexural and diagonal shear cracks were observed. The first flexural crack appeared at a load of 103.8 kN, followed by a diagonal crack at 122 kN, approximately 145 mm from the support, oriented at nearly 45°. This behavior is typical of combined shear–flexure failure in relatively deep beams.

The un-strengthened beam with a 150 mm circular web opening (B2) exhibited earlier diagonal cracking, initiating at only 48% of the control beam’s cracking load. The crack started from the lower edge of the opening and propagated diagonally toward the loading point. No flexural cracks were detected due to the significant stiffness reduction caused by the opening. This specimen failed prematurely, carrying only 32% of the control beam’s ultimate load, which highlights the detrimental impact of large web openings on both strength and ductility.

Beams retrofitted internally with steel fibers (B4 and B5) showed improved cracking resistance and ultimate load capacity. The inclusion of 0.5% and 1.0% steel fibers raised the first cracking load to 50% and 56% of the control beam, respectively. This improvement is attributed to the fibers bridging micro cracks and delaying their propagation.

For fire-exposed beams with steel fibers (B6 and B7), cracks developed during the heating phase under a sustained load of 30 kN. After cooling, specimen B7 exhibited a vertical flexural crack at 40 kN, while B6 showed no flexural cracking. Both beams, however, demonstrated superior behavior compared to un-strengthened fire-exposed specimens, with ultimate loads increasing by 6.8% (0.5% fibers) and 16.4% (1.0% fibers) relative to the fire-damaged reference. These results confirm that steel fibers provide effective post-fire crack control and shear resistance by confining damage and sustaining load capacity.

Externally retrofitted beams (B8 and B9), strengthened with ferrocement jackets, also performed better than their un-strengthened counterparts. In the unheated case (B8), the first cracking load reached 54% of that of the control beam. After fire exposure, specimen B9 experienced similar cracking loads, indicating partial thermal degradation of the mortar. Cracks were concentrated near the openings, extending diagonally toward the jacket edges. In B8, flexural cracks initiated at 80 kN and stabilized around 100 kN, while B9 did not exhibit visible flexural cracks.

The ferrocement jackets remained well bonded throughout the tests, effectively limiting crack growth. Compared to the un-strengthened fire-damaged beam (B3), the ultimate loads of B8 and B9 were higher by approximately 10% and 14%, respectively. These findings demonstrate the effectiveness of ferrocement as an external retrofitting technique for enhancing both fire resistance and load-bearing capacity of HSC beams with web openings.

### Load–deflection curves

#### Effect of openings and fire exposure on load–deflection response

The experimental load–deflection curves (Fig. 4) demonstrated a pronounced reduction in the structural performance of beams containing circular openings, particularly in terms of stiffness, ductility, and energy absorption. In the initial elastic stage, all specimens exhibited similar slopes, indicating comparable stiffness. However, once the first crack appeared, a sudden increase in mid-span deflection was observed, marking the onset of damage.

The un-strengthened beam with a 150 mm circular opening (B2) experienced the most severe deterioration. Its ultimate load reached only 216 kN, which corresponds to 32% of the control specimen (B1). In addition, the maximum deflection at failure decreased to 8.11 mm compared to 15.18 mm in the control beam (Table 2), representing a 47% reduction. This confirms the adverse influence of large openings on both strength and deformability.

When exposed to fire, beams were subjected to a constant preload of 30 kN during the heating stage. Under this condition, the mid-span deflection progressively increased to 3.08 mm. Following cooling by water spraying, the deflection decreased to 1.58 mm, reflecting a 48.7% recovery due to partial stiffness regain after thermal contraction. Nevertheless, degradation of the concrete matrix was evident. Upon subsequent loading, the deflection rose to 4.75 mm at peak load and reached 4.93 mm at ultimate failure, in agreement with the reduced ductility indices summarized in Table 2.

Overall, the results highlight that the combination of circular openings and fire exposure leads to substantial reductions in stiffness, load-carrying capacity, and ductility. Specifically, the ductility index decreased from 1.67 in the solid control beam (B1) to 1.32 in the fire-damaged specimen with an opening (B3). This trend emphasizes the vulnerability of beams with openings under elevated temperatures.

#### Effect of retrofitting with discrete steel fibers

The incorporation of discrete steel fibers as an internal retrofitting technique substantially enhanced the structural response of beams with circular openings, particularly under fire exposure. In the unheated condition, beams containing 0.5% and 1.0% steel fibers (B4 and B5) achieved ultimate loads of 228 kN and 237 kN, respectively, compared with only 216 kN for the un-strengthened specimen with an opening (B2). After fire exposure, the ultimate capacities of the fiber-reinforced beams (B6 and B7) further increased to 187 kN and 203 kN, representing improvements of 6.8% and 16.0%, respectively, relative to the fire-damaged un-strengthened beam (B3) (Table 1).

The elastic stiffness of fiber-reinforced beams remained similar to that of the un-strengthened counterparts. However, in the post-cracking range, the load–deflection curves exhibited a more gradual decline, reflecting improved crack control and greater energy dissipation (Fig. 5). The maximum deflection decreased significantly, from 7.12 mm in B3 to 7.02 mm in B6 and 5.96 mm in B7, corresponding to reductions of 1.4% and 16.3%, respectively (Table 2). Nevertheless, the ductility indices of fiber-reinforced beams under fire remained between 1.24 and 1.29, indicating only marginal enhancement in deformation capacity.

These results demonstrate that discrete steel fibers are highly effective in restoring load-carrying capacity and controlling crack propagation in beams with openings, even after thermal degradation. However, their contribution to improving global ductility under combined thermal and mechanical loading is limited (Fig. 5).

#### Effect of ferro cement retrofitting on beam deflection

The application of external ferrocement layers considerably enhanced the structural response of beams with circular openings, both under ambient and elevated temperature conditions. Under normal conditions, the retrofitted specimen B8 achieved an ultimate load of 237.5 kN, representing a 10% improvement compared to the un-strengthened beam with an opening (B2 = 216 kN). After fire exposure, specimen B9 sustained an ultimate load of 200.9 kN, which is 14.5% higher than the fire-damaged un-strengthened beam (B3 = 175 kN) (Table 1).

The deformation behavior also reflected a significant improvement. The maximum deflection at peak load decreased from 7.12 mm in B3 to 4.93 mm in B9, representing a 30.7% reduction. Similarly, under ambient conditions, the deflection of B8 was limited to 3.52 mm, which is only 23% of the control beam (B1 = 15.18 mm) (Table 2). The ductility index of ferrocement-retrofitted specimens remained between 1.13 and 1.71, indicating moderate enhancement in post-cracking deformation capacity. Crack development in ferrocement-retrofitted beams was characterized by the formation of multiple fine cracks instead of fewer wide cracks, confirming the superior crack-arresting capability of ferrocement layers (Fig. 6). This confinement effect was particularly beneficial after fire exposure, where the ferrocement jacket remained intact and well-bonded, preventing sudden brittle failure. However, the strengthening efficiency was less pronounced in beams with large openings located within shear-critical zones, suggesting that ferrocement retrofitting alone cannot fully compensate for severe reductions in stiffness caused by wide penetrations.

The experimental outcomes confirm the pronounced effect of fire exposure at 500 °C on beams with circular openings, which suffered considerable reductions in load capacity, ductility, and stiffness when left un-strengthened.

Retrofitting with discrete steel fibers enhanced the post-fire behavior, particularly in terms of toughness and energy absorption. As indicated in Table 2, the area under the load–deflection curve of the 1.0% fiber-reinforced beam (B7) was 1159, representing a 15% improvement compared with the fire-exposed un-strengthened specimen (B3 = 1005). This reflects the fibers’ ability to sustain load beyond initial cracking. However, the ductility index of fiber-reinforced beams under fire exposure (1.24–1.29) remained lower than the un-strengthened beam (1.32), suggesting that the fibers were less effective in maintaining deformability under elevated temperatures.

On the other hand, ferrocement retrofitting proved more effective in controlling deflections and enhancing stiffness. The fire-exposed ferrocement specimen (B9) exhibited a maximum mid-span deflection of 4.93 mm, which is 31% lower than the un-strengthened fire-damaged beam (B3 = 7.12 mm), as illustrated in Fig. 6**.** The reduction of area also decreased significantly, confirming the ability of ferrocement jackets to confine cracks and preserve sectional integrity under thermal stress. Nevertheless, the ductility index of ferrocement-retrofitted specimens (1.13–1.71) was generally lower than that of fiber-reinforced beams under ambient conditions, reflecting the more stiffness-dominated strengthening effect of ferrocement.

In summary, steel fibers are more effective for improving energy absorption and post-cracking toughness, whereas ferrocement retrofitting excels in reducing deflections and improving stiffness under fire exposure. The selection of retrofitting technique should therefore be based on the target performance requirement: higher ductility (fibers) or enhanced stiffness and deformation control (ferrocement).

## Numerical analysis

Using the commercial program ABAQUS, finite element modeling (FEM) was used to numerically examine the flexural behavior of nine high-strength reinforced concrete (HSC) beams^26,27,25^. The analysis’s objectives were to (i) determine how effectively beams with circular web openings performed in temperatures, and (ii) determine how effectively internal and external retrofitting methods worked. To ensure accuracy and dependability, numerical models were created to replicate the experimental specimens, and the results were verified and calibrated against experimental observations.

### Finite element modeling approach

**Fig. 7:**
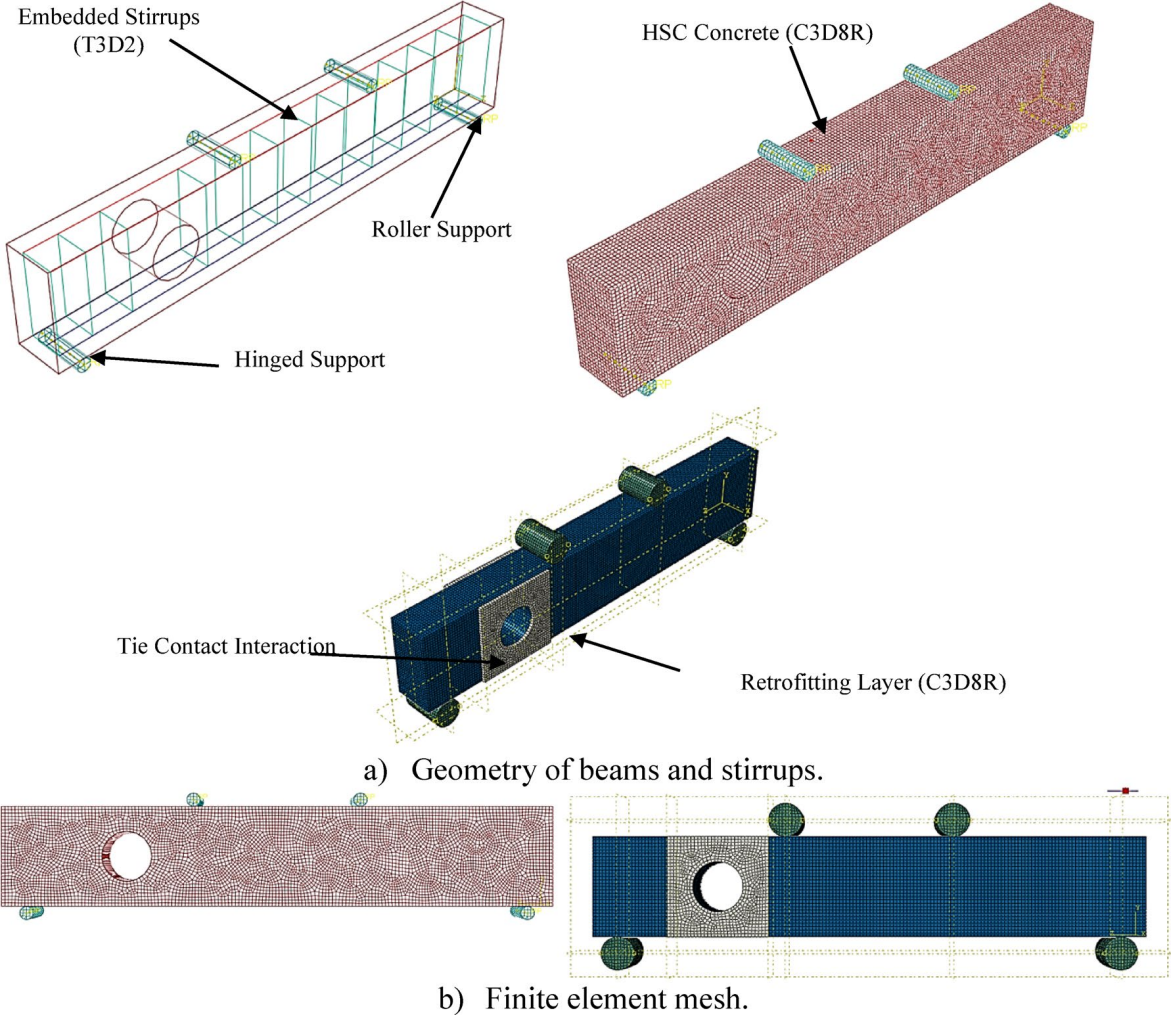
Details of the numerical model.

As shown in Fig. 7a, the simulation included beam geometry, including the HSC section and retrofitting systems. Additionally, loading and support plates were modeled. Hourglass control and 8-node linear brick elements with reduced integration were used to discretize concrete beams, retrofitting layers, and plates (C3D8R). Two-node linear three-dimensional truss elements (T3D2) were used to represent reinforcement bars, such as stirrups and longitudinal steel. A consistent mesh size of 10 mm was chosen to strike a balance between solution accuracy and computational efficiency.

A tie constraint was used to simulate the monolithic casting process between the beams and retrofitting layers, and the “embedded region” technique was used to define the interaction between concrete and reinforcement. In accordance with the experimental configuration, boundary conditions were modeled as a pinned support at the right end and a roller support at the left. The loading plates were subjected to controlled displacement to apply flexural loading.

### Material properties and constitutive models

Material properties were defined based on experimental stress–strain results (Fig. 7b). The Poisson’s ratios were taken as 0.18 for HSC and 0.20 for the ferrocement mesh. Young’s modulus values were derived from the initial linear portion of the stress–strain curves. The nonlinear behavior of concrete was modeled using the Concrete Damaged Plasticity (CDP) model available in ABAQUS^22,23^. The CDP parameters are summarized in Table 3.

**Table 3:** Parameters of the CDP model for HSC and retrofitting layers.

Concrete type	Dilation angle (ψ)	Eccentricity (e)	Shape parameter (K_c_)	Maximum compression axial/biaxial (*f*_*bo*_/*f*_*co*_)	Viscosity (μ)
HSC	35	0.1	2/3	1.16	0
Retrofitting layers	40	0.1	2/3	1.16	0

The reinforcing steel was modeled as an elastic–perfectly plastic material with an elastic modulus of 200 GPa. Longitudinal reinforcement bars had a yield stress of 400 MPa, while transverse stirrups (8 mm diameter) had a yield stress of 240 MPa.

### Thermal properties under elevated temperatures

The fire analysis incorporated the thermal, mechanical, and thermo-mechanical properties of concrete and steel, which govern heat transfer and structural behavior under high temperatures. For concrete, temperature-dependent properties included thermal conductivity (k), specific heat capacity (c), density (ρ), and coefficient of thermal expansion (α). For steel, thermal conductivity (ks), specific heat (cs), and coefficient of expansion (αs) were considered. These values were adopted from ISO-834 (1999) and Eurocode 2 provisions, as summarized in Table 4.

**Table 4:** Thermal properties of concrete and steel at elevated temperatures.

Temperature	Steel		concrete		
	specific heat	conductivity	density	specific heat	conductivity
20	439.8	53.334	2400	900	1.333
100	487.62	5.07E + 01	2400	900	1.2297
200	529.76	47.34	2352	1000	1.1108
300	564.74	44.01	2316	1050	1.0033
400	605.88	40.68	2280	1100	0.9072
500	666.5	37.35	2244	1100	0.8225

### Numerical results and validation

**Fig. 8:**
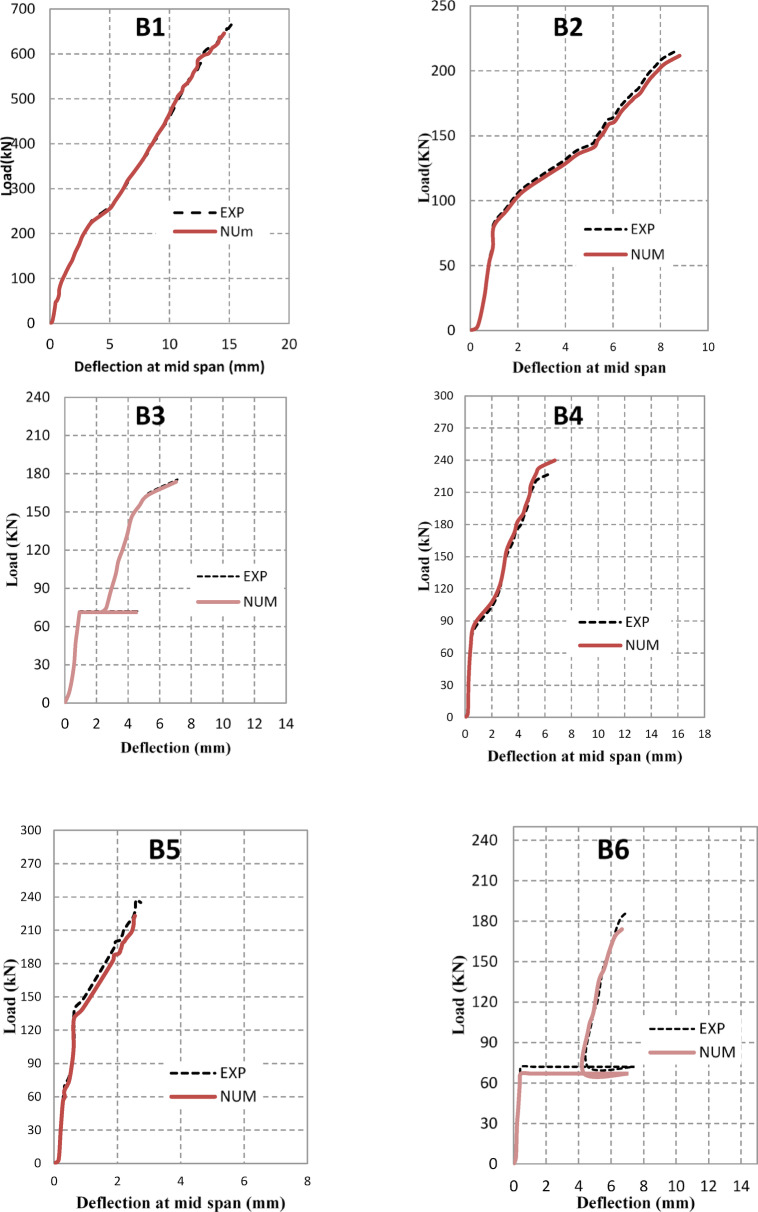

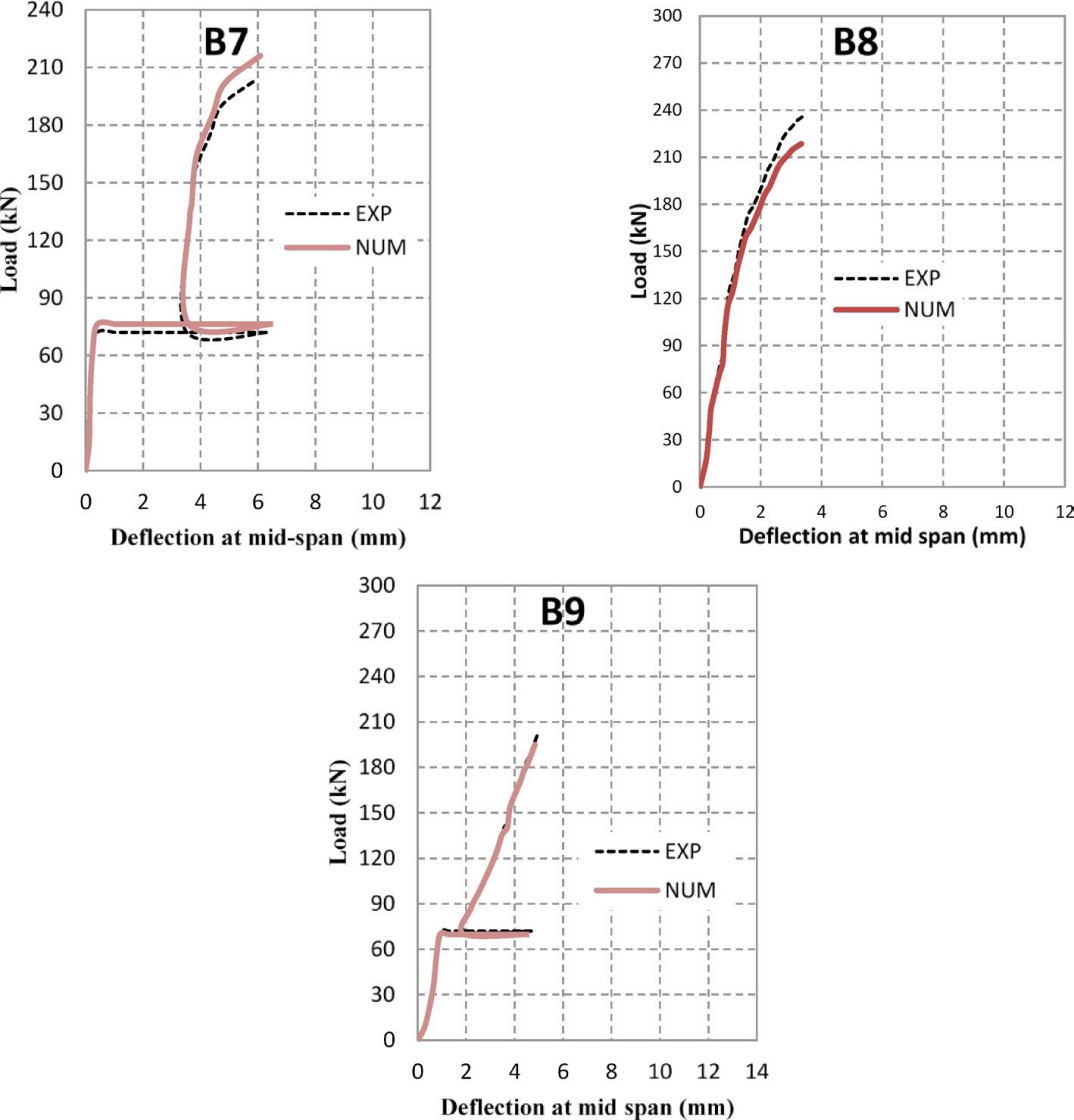
Experimental Vs numerical load–deflection behavior for beams.

The FEM successfully captured the nonlinear flexural response, progressive cracking, and layered interaction of the beams under elevated temperatures. Figure 8 compares experimental and numerical load–deflection responses, while Table 5 summarizes ultimate load (Pu) and ultimate deflection (Δu) values. In addition to experimental validation, parametric numerical analyses were conducted using the validated FEM model to investigate the influence of opening diameter (100–200 mm) and elevated temperature levels (400–600°C) on the shear behavior of HSC beams. The numerical model incorporated temperature-dependent material properties, nonlinear concrete damaged plasticity (CDP), and thermal expansion effects, as summarized in Table 4.

**Table 5:** Comparison of the experimental and numerical results.

Group	Specimen	P_u_ (KN)				Δ_Pu_(mm)		
		Exp	Num	Exp./ Num	Exp		Num	Exp./Num
G1	B1	666	646	1.03	15.18		14.58	1.04
	B2	216	211	0.98	8.11		8.19	0.99
	B3	175	173.6	1.008	7.125		7.001	1.018
G2	B4	228	239	0.95	6.55		6.74	0.97
	B5	237	223	1.06	2.61		2.56	1.02
	B6	187	174	1.07	7.02		6.67	1.05
	B7	203	216	0.94	5.96		6.08	0.98
G3	B8	237.5	219	1.08	3.52		3.34	1.05
	B9	200.87	195	1.03	4.935		4.836	1.02

The results confirm the experimentally observed trends, showing progressive shear degradation with increasing opening size and temperature. Furthermore, the analyses demonstrate that internal steel fiber reinforcement and external ferrocement jacketing remain effective in mitigating post-fire shear capacity loss, particularly for moderate opening diameters.

**Fig. 9:**
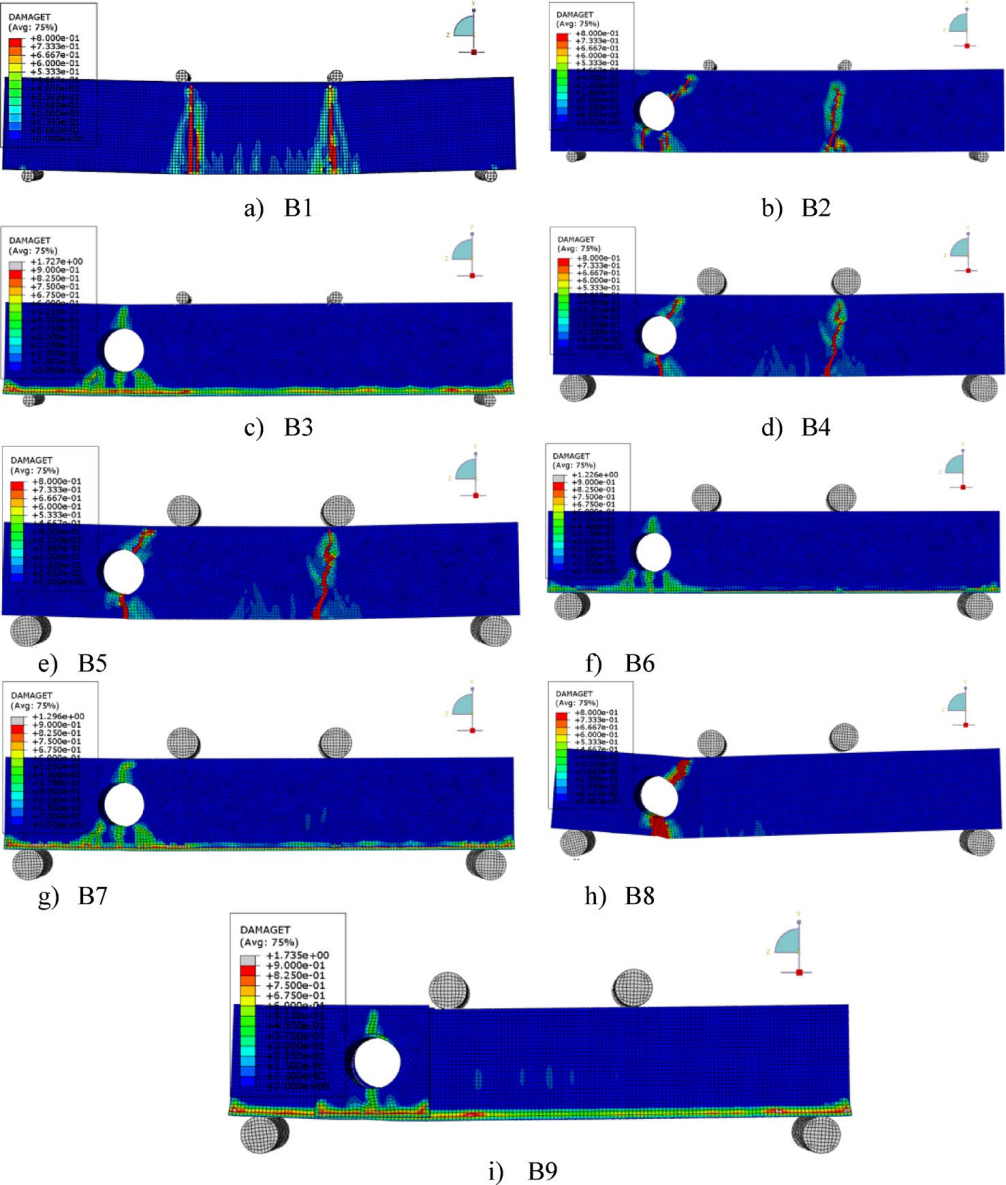
FEM failure crack and failure pattern of beams.

The FEM results also reproduced the failure modes and crack propagation patterns with strong agreement to experimental observations, as illustrated in Fig. 9.

The average ratio of experimental to numerical ultimate loads was 1.02, while the average ratio for ultimate deflection was 1.03, confirming the high accuracy of the FEM predictions. Figure 8 further illustrates the strong agreement between experimental and numerical crack patterns, where FEM successfully reproduced diagonal shear cracks around the openings and confinement effects in retrofitted beams.

Overall, the FEM model demonstrated reliable predictive capacity, validating its use for parametric studies on the influence of opening size, fire exposure, and strengthening strategies on the flexural behavior of HSC beams.

Although the current study investigates a representative set of HSC beams, future work should explore parametric variations in opening diameter, reinforcement ratios, and fire exposure durations to generalize the findings. The updated finite element model, incorporating temperature-dependent constitutive laws, provides a robust framework for such extended analyses.

## Conclusions

This study comprehensively investigated the structural performance of high-strength reinforced concrete (HSC) beams with circular web openings under fire exposure and evaluated the effectiveness of both internal and external retrofitting strategies. Based on the experimental and theoretical findings, the following conclusions are drawn:

1. The presence of large circular web openings led to a substantial reduction in shear capacity and stiffness of high-strength RC beams. Under fire exposure at 500 °C, the shear capacity decreased by up to 68% compared with solid control specimens.
2. Internal retrofitting using steel fibers (0.5%–1.0% by volume) enhanced post-fire shear resistance and crack control, resulting in a recovery of shear capacity of up to **16.4%** relative to un-retrofitted fire-damaged beams.
3. External ferrocement jacketing improved stiffness and reduced mid-span deflections, achieving a shear capacity recovery of up to 14.5% after fire exposure.
4. The validated FEM model, incorporating temperature-dependent material properties, demonstrated strong agreement with experimental results, with an average deviation not exceeding 3%.
5. Parametric numerical analyses indicated that increasing opening diameter and fire temperature significantly amplify shear degradation, while retrofitting efficiency diminishes for openings larger than 200 mm.
6. The findings provide practical insight into the post-fire assessment and retrofitting of high-strength RC beams with web openings, while acknowledging that additional experimental studies are required to generalize the results.

## Data Availability

“The datasets generated and/or analyzed during the current study are available from the corresponding author on reasonable request.”
